# Trends and Hospital Outcomes in HOSPITAL Admissions for Anaphylaxis in Children with and without Asthma in Spain (2016–2021)

**DOI:** 10.3390/jcm12196387

**Published:** 2023-10-06

**Authors:** Javier De Miguel-Díez, Ana Lopez-de-Andres, Francisco J. Caballero-Segura, Rodrigo Jimenez-Garcia, Valentin Hernández-Barrera, David Carabantes-Alarcon, Jose J. Zamorano-Leon, Ricardo Omaña-Palanco, Natividad Cuadrado-Corrales

**Affiliations:** 1 Respiratory Department, Hospital General Universitario Gregorio Marañón, Facultad de Medicina, Instituto de Investigación Sanitaria Gregorio Marañón (IiSGM), Universidad Complutense de Madrid, 28007 Madrid, Spain; javier.miguel@salud.madrid.org (J.D.M.-D.); fcabal01@ucm.es (F.J.C.-S.); 2 Department of Public Health and Maternal & Child Health, Faculty of Medicine, Universidad Complutense de Madrid, 28040 Madrid, Spain; rodrijim@ucm.es (R.J.-G.); dcaraban@ucm.es (D.C.-A.); josejzam@ucm.es (J.J.Z.-L.); romana@ucm.es (R.O.-P.); mariancu@ucm.es (N.C.-C.); 3 Preventive Medicine and Public Health Teaching and Research Unit, Health Sciences Faculty, Universidad Rey Juan Carlos, 28922 Alcorcón, Spain; valentin.hernandez@urjc.es

**Keywords:** anaphylaxis, children, hospitalizations, trends, asthma, sex-differences

## Abstract

(1) Background: To assess and compare the temporal trends in the incidence, characteristics and hospital outcomes among children with and without asthma who were hospitalized with anaphylaxis in Spain from 2016 to 2021, and identify the variables associated with severe anaphylaxis among children with asthma. (2) Methods: An observational, retrospective study was conducted using a population-based database. The study population included pediatric patients with anaphylaxis. This population was stratified based on whether they had asthma. (3) Results: The number of hospital admissions was stable from 2016 to 2019, dropping in 2020 and raising to the highest number in 2021. A total of 60.63% of hospitalizations occurred in boys and the most common anaphylactic reactions were due to food consumption (67.28%), increasing over time. The in-hospital mortality (IHM) remained stable and under 1% in all the years studied. The incidence of anaphylaxis was 2.14 times higher in children with asthma than in those without asthma (IRR 2.14; 95% CI 1.87–2.44). Furthermore, it was 1.79 times higher in boys with asthma than in those without asthma (IRR 1.79; 95% CI 1.06–2.45) and 2.68 times higher in girls with asthma than in those without asthma (IRR 2.68; 95% CI 2.23–3.12). Asthma was not associated with severe anaphylaxis (OR 1.31; 95% CI 0.88–1.96). (4) Conclusions: The number of hospitalizations for anaphylaxis in children remained stable from 2016 to 2019, dropping in 2020 and recovering in 2021. IHM was low and remained stable during the study period. The incidence of hospitalizations for anaphylaxis was higher in asthmatic children than in non-asthmatics, but there were no differences in the occurrence of severe anaphylaxis among them.

## 1. Introduction

Anaphylaxis is a serious, potentially life threatening, allergic reaction that is triggered suddenly by exposure to specific allergen substances [1,2,3,4]. It may occur at any age and often results in hospital and/or emergency department admissions [5]. For this reason, healthcare professionals require training in how to recognize anaphylaxis and differentiate it from other diagnoses [6]. The lifetime prevalence of anaphylaxis is around 5% and appears to be rising [7]. In children, the estimated prevalence ranges from 0.04% to 1.8% and is also increasing, mostly at pre-school age [8].

Epidemiological factors associated with anaphylaxis vary with age, culture and lifestyle [9]. Food has been considered the predominating cause of anaphylaxis, particularly among children, and represents a significant health problem, as anaphylaxis caused by food can contribute to mortality [10].

Between 11% and 38% of children who experienced anaphylaxis have a history of asthma or recurrent wheezing [11]. Asthma has been identified as a risk factor for severe and potentially fatal anaphylaxis [12]. However, in a recent study, children hospitalized for anaphylaxis with a history of asthma did not have a higher chance of severe anaphylactic reactions compared with children without asthma [13].

Despite the substantial burden that this problem produces in the pediatric population, data concerning the characteristics of anaphylaxis in children in Spain are spare and they are not updated [14,15]. Studies on temporal trends based on large national admission databases could help to address this knowledge gap. The objectives of our study were as follows: (a) to assess the temporal trends in the incidence, anaphylactic reaction triggers, clinical characteristics, and hospital outcomes among children with and without asthma who were hospitalized with anaphylaxis in Spain from 2016 to 2021; (b) to compare study variables between children with and without asthma stratified by age and sex; (c) to identify variables associated with severe anaphylaxis, defined as causing admission to the intensive care unit (ICU) and/or mortality during hospitalization, among children with asthma, and to evaluate the effect of asthma on the occurrence of severe anaphylaxis.

## 2. Materials and Methods

An observational, retrospective study (from 1 January 2016 to 31 December 2021) was conducted using a population-based database, the Minimum Basic Data Set of Specialized Care Activity Registry (RAE-CMBD in Spanish). The RAE-CMBD, owned by the Spanish Ministry of Health (SMH), records individual information of all patients admitted to Spanish hospitals. The collected information includes sex, age, admission date, discharge date, diagnoses (up to 20), procedures (up to 20), and discharge destination (voluntary discharge, home, social institution, deceased) [16]. The RAE-CMBD uses the International Classification of Diseases, Tenth Revision (ICD-10) for coding. All diagnosis and procedure codes used in this study are detailed in Table S1.

The study population included pediatric patients (17 years old or younger) with an ICD-10 code for anaphylaxis in any diagnostic position within the RAE-CMBD. Subsequently, this population was stratified based on whether they had an asthma code in any diagnostic position within the RAE-CMBD. Hospitalized patients with lacking data for age, sex, dates of admission and/or discharge and discharge destination were excluded.

Different types of anaphylactic reaction triggers were recorded: food, drugs, serum, or unspecified, according to the ICD-10 coding methodology.

The use of mechanical ventilation (invasive and non-invasive), codified in any position in the RAE-CMBD procedure field, was also analyzed (Table S1).

Admission to the ICU refers to being admitted to the intensive care unit during hospitalization with anaphylaxis for at least 24 h. In-hospital mortality (IHM) was the proportion of deaths during hospitalization. A child with severe anaphylaxis was defined as someone who required admission to the ICU and/or died during hospitalization.

### 2.1. Statistical Analysis

The incidence of hospitalization with anaphylaxis per 100,000 children with and without asthma was calculated for each of the six years analyzed. Incidence rates were calculated based on the Spanish pediatric population with asthma, grouped by age and sex, according to the National Health Survey of 2016/2017 [17]. The pediatric populations with asthma for the missing years (2018, 2019, 2020, and 2021) were estimated assuming that the growth rate remained stable throughout the period. Poisson regression was used to calculate age- and sex-adjusted incidence rate ratios (IRR) with their 95% confidence intervals (CI).

A descriptive statistical analysis was conducted, categorical variables were expressed as frequencies and percentages, and for quantitative variables, mean and standard deviation were provided.

Temporal trends were analyzed using Cochran–Mantel–Haenszel statistics or the Cochran–Armitage test for categorical variables, and linear regression *t*-test or Jonckheere-Terpstra test for continuous variables.

Categorical variables were compared using Fisher’s exact test, and continuous variables were compared using *t*-test or Mann–Whitney test, as necessary.

Multivariable logistic regression was used to identify variables associated with severe anaphylaxis among children with anaphylaxis according to asthma status. The models were constructed, including sex, age, year, reaction triggers and use of mechanical ventilation. The results of these models are presented as odds ratios (OR) with 95% CI.

Statistical analysis was performed using Stata version 14 software (Stata, College Station, TX, USA). A *p*-value of <0.05 (two-tailed) was considered significant.

### 2.2. Ethical Statement

According to Spanish legislation, written consent from patients or evaluation by an ethics committee is not required since the RAE-CMBD is an administrative, mandatory, ammonized registry, and its data can be requested online to the SMH [18]. The SMH will evaluate the ethical and methodological issues of the suggested study protocol and, if considered adequate, will provide the database.

## 3. Results

As can be seen in Table 1, during the study period, 2016–2021, there were 2573 hospital admissions with anaphylaxis in children under 18 years old in Spain.

Of all the hospital admissions, 60.63% occurred in boys, with a mean age of the study population of 6.77 years.

The most common anaphylactic reactions were due to food consumption (67.28%), followed by drug consumption (12.98%) and serum consumption (3.03%). Unspecified reactions were codified in 16.95% of the cases.

The need for mechanical ventilation was infrequent, with overall values of 2.68% for invasive and 1.32% for noninvasive, respectively.

Among the children diagnosed with anaphylaxis from 2016 to 2021, 10.57% were admitted to the ICU, and the IHM rate was 0.66%. Severe anaphylaxis, defined as either admission to the ICU or IHM, occurred in 10.77% of the children.

### 3.1. Temporal Trends in Hospital Admissions with Anaphylaxis in the Spanish Pediatric Population between 2016 and 2021

As indicated in Table 1, the number of hospital admissions was stable from 2016 to 2019 (around 430 per year) dropping to 346 in 2020 and rising to the highest number in 2021 (504).

**Figure 1:**
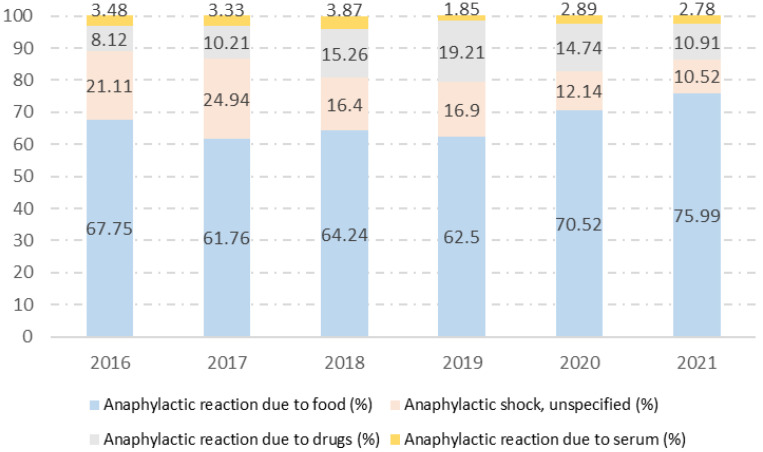
Triggers of anaphylaxis hospitalizations among Spanish children from 2016 to 2021.

Between 2016 and 2021, there was a significant increase in reactions associated with food consumption (67.75% vs. 75.99%; *p* < 0.001) and reactions associated with drug consumption (8.12% vs. 10.91%; *p* < 0.001). However, non-specific reactions decreased over the study period (21.11% vs. 10.52%; *p* < 0.001). Figure 1 shows the evolution over time according to the trigger type.

The use of non-invasive mechanical ventilation increased in children with anaphylactic reactions between 2016 and 2021 (0.46% vs. 2.18%; *p* = 0.021), as observed in Table 1.

The IHM remained stable and under 1% in all the years studied. The ICU admissions and the prevalence of severe anaphylaxis showed significant variations over the study period. oscillating between 6.73% in 2016 and 13.66% in 2019.

### 3.2. Temporal Trends and Characteristics of Hospital Admissions with Anaphylaxis in the Spanish Pediatric Population Based on the Presence of Asthma

The presence of asthma among children admitted with anaphylaxis from 2016 to 2021 was codified in 12.59% (n = 324) of cases and remained stable overtime (Table 2).

Children with asthma had a higher mean age than children without asthma (9.85 years vs. 6.33 years; *p* < 0.001) and a similar sex distribution.

Anaphylactic reactions due to food consumption (73.46% vs. 66.39%; *p* = 0.011) were more frequent among children with asthma, but fewer reactions due to drug consumption (7.1% vs. 13.83%; *p* < 0.001) were found, as shown in Table 2.

**Figure 2:**
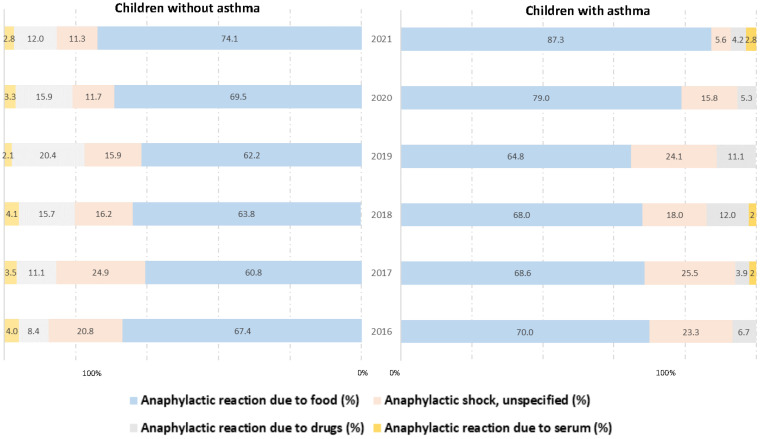
Triggers of anaphylaxis hospitalizations among Spanish children from 2016 to 2021, according to asthma status.

Both in children with and without asthma, reactions associated with food consumption significantly increased over the study period (70% and 67.39% in 2016 vs. 87.32% and 74.13% in 2021; *p* = 0.044 and *p* < 0.001, respectively). Reactions associated with drug consumption increased in the group of non-asthmatic children (8.36% in 2016 vs. 12.01% in 2021). However, non-specific anaphylactic reactions decreased in both asthmatic children (23.33% in 2016 vs. 5.63% in 2021; *p* = 0.037) and non-asthmatic children (20.75% vs. 11.32%; *p* < 0.001). Figure 2 shows the triggers of anaphylaxis among Spanish children with and without asthma from 2016 to 2021

The use of non-invasive mechanical ventilation significantly increased (*p* = 0.018) in the group of non-asthmatic children between 2016 and 2021, as indicated in Table 2.

In non-asthmatic children, the frequency of ICU admissions increased over the study bperiod (*p* = 0.004). However, the frequency of severe anaphylaxis decreased (*p* = 0.006). In children with asthma, over the study period, ICU admissions, in-hospital mortality, and the frequency of severe anaphylaxis remained stable, as shown in Table 2.

### 3.3. Incidence of Hospital Admission Due to Anaphylactic Reaction According to the Presence of Asthma and Characteristics of Admission According to Age and Sex

Table 3 presents the characteristics of hospital admissions with anaphylaxis by age groups and according to the asthma status.

The crude incidence of hospital admission with anaphylaxis in patients with asthma was 59.75 cases per 100,000 subjects with asthma per year, while in patients without asthma, it was 27.98 cases. Among patients with asthma, the highest incidence was observed in the age group of 6–11 years (82.56), whereas in patients without asthma, it was in the age group between 0 and 5 years (45.48). After using the Poisson regression model, adjusted for age and sex, it was found that the incidence of anaphylaxis for the period 2016–2021 was 2.14 times higher among children with asthma than among those without asthma (IRR 2.14; 95% CI 1.87–2.44).

When comparing the characteristics of anaphylaxis admission based on age groups, patients without asthma, aged between 12 and 17 years, had fewer reactions associated with food consumption and more reactions associated with drug consumption and non-specific reactions (all *p* < 0.001) compared to the other age groups (0–5 years old and 6–11 years old). Additionally, they had more admissions to the ICU (*p* = 0.001) and more severe anaphylactic reactions (*p* = 0.002), as indicated in Table 3.

Table 4 presents the characteristics of hospital admissions with anaphylaxis by sex and according to the asthma status.

The incidence of hospital admission with anaphylaxis in boys with asthma was 60.19 cases per 100,000 subjects with asthma, while in boys without asthma, it was 33.37. In girls, the incidence was 59.14 in those with asthma and 22.33 in those without asthma. After conducting the Poisson regression model, the incidence of anaphylaxis was 1.79 times higher in boys with asthma than in those without asthma (IRR 1.79; 95% CI 1.06–2.45) and 2.68 times higher in girls with asthma than in those without asthma (IRR 2.68; 95% CI 2.23–3.12).

When comparing admission characteristics by sex, it was observed that in those without asthma, the mean age of girls was higher than that of boys (6.7 years vs. 6.09 years; *p* = 0.004). Additionally, girls had significantly fewer reactions associated with food consumption (63.28% vs. 68.37%; *p* = 0.013). In patients with asthma, only the use of non-invasive mechanical ventilation was higher in boys than in girls (*p* = 0.035). The rest of the study variables presented similar values between boys and girls, as indicated in Table 4.

### 3.4. Multivariable Analysis of Factors Associated with Severe Anaphylaxis during Hospital Admission with Anaphylaxis in the Pediatric Population with and without Asthma

As indicated in Table 5, older age (12–17 years) was a risk factor for severe anaphylaxis in children without asthma (OR 1.48; 95% CI 1.02–2.15).

The presence of reactions associated with drug consumption was associated with severe anaphylaxis in children without asthma and in the total study population.

Additionally, in the total study population, undergoing invasive and non-invasive mechanical ventilation (OR 17.54; 95% CI 9.87–31.15 and OR 4.9; 95% CI 2.07–11.6, respectively) were risk factors associated tor severe anaphylaxis.

After adjustment, severe anaphylaxis was significantly higher in the study population in years 2017, 2018, 2019, and 2020 compared to the year 2016.

Finally, when using the non-asthma status as the reference category, the analysis of the entire database revealed that the presence of asthma was not associated with severe anaphylaxis (OR 1.31; 95%CI 0.88–1.96).

## 4. Discussion

Our study provides new data regarding anaphylaxis among children. We reported that the number of hospital admissions for anaphylaxis in children remained stable from 2016 to 2021. Previous studies investigating trends in anaphylaxis hospitalization among children have reported conflicting results. Agreeing with us, Robinson et al. [19] found that anaphylaxis hospitalizations among infants and toddlers in the United States were stable from 2006 to 2015, in contrast to rising trends in older children. Similarly, Shrestha et al. [20] reported a stable rate of anaphylaxis hospitalizations among children and adults in the United States from 2001 to 2014, finding only an increase in children aged 5 to 14 years with food-related reactions.

Declining trends have been reported by Motosue et al. [21] who showed a significant decline in hospitalizations for United States children presenting with food-induced anaphylaxis from 2005 to 2014, despite a rise in emergency department visits.

Finally, increments in hospitalizations have been published by Dyer et al. [22], who described rising rates of food-induced anaphylaxis hospitalizations and emergency department visits in children in Illinois from 2008 to 2012. Also, Tejedor–Alonso et al. [14] reported an increase in the frequency of admission due to anaphylaxis in Spanish hospitals from 1998 to 2011, particularly in patients aged 0–14 years and in food anaphylaxis. More recently, Baseggio Conrado et al. [23] evidenced a threefold increase in hospital admissions for food anaphylaxis between 1998 and 2018 in the United Kingdom, with cow’s milk being the most common single cause of fatal anaphylaxis.

The underlying reasons for the stable trends in anaphylaxis hospitalizations in children obtained in our study are not known. We do not believe that our findings are due to a decline in prevalence of anaphylaxis. In fact, recent studies on the trends in prevalence of anaphylaxis and food allergy support a rising prevalence [5]. It is likely that our findings are the results of several factors, including changes in disease recognition, severity, management, and health care utilization, as already described by other authors [19,24,25].

We also found a drop in the number of hospital admissions for anaphylaxis in 2020, coinciding with the start of the COVID pandemic, which was recovered in 2021. The decrease in the frequency of anaphylaxis at the start of the pandemic has been described by other authors [26], as well as the subsequent recovery [27]. It may reflect decreased accidental exposures due to reduced social gatherings and closed schools. The reluctance to present to the emergency department for fear of contagion may also contribute [26,27].

Our data showed that anaphylaxis hospitalization was more likely in male than in female children. These results are consistent with those reported by the majority of previous studies [28,29]. Regardless of gender, we also found that food was the most common cause of anaphylaxis in children, as previously described [30,31], representing more than two thirds of the cases. Furthermore, unspecified causes decreased, while reactions caused by food and drugs increased over time, possibly influenced, at least in part, by improved coding.

Severe anaphylaxis requiring ICU admission is a rare event and difficult to study since the number of affected patients is usually small [32]. We identified that 10.57% of children with anaphylaxis required admission to the ICU. Sundquist et al. [33] also found a lower ICU admission rate in children with anaphylaxis, which is clearly less than in adults.

Consistent with other studies [34], we found that IHM was low and remained stable over time. So, the vast majority hospital admissions with anaphylaxis did not result in death, reflecting in part the quality-of-care provided [35]. In fact, mortality appears similar in those regions where data are available [24]. An exception is Australia, where all-cause fatal anaphylaxis rates increased by 6.2% per annum from 1997 to 2013, primarily due to food triggers [36]. However, when these data are analyzed by case-fatality rate (proportion of cases admitted to hospital that result in a fatal outcome), mortality has decreased, including with respect to fatal food-related anaphylaxis in Australia [24].

Asthma seems to be associated with the risk of anaphylaxis. In the current study, the incidence of hospitalizations for anaphylaxis was higher in asthmatic children than in non-asthmatic children. González–Pérez et al. [37] also demonstrated that patients with asthma have a greater risk of anaphylaxis than those without asthma, with the risk greater in severe than no severe asthma. Like us, they also found that women are at higher risk of anaphylaxis than men, especially if they have severe asthma.

The relationship between asthma and severe anaphylaxis is controversial. Despite asthma having been identified as a risk factor for severe anaphylaxis [38,39,40,41], our study did not find that children with asthma had more severe anaphylaxis compared with those without asthma. Similar results have been described by other authors [11,13,42]. Furthermore, Motosue et al. [43] reported that asthma was less likely to be a predictor of hospitalizations, admissions to the ICU, and endotracheal intubation. However, other authors have indicated that suboptimal asthma control, rather than the presence of asthma, may increase the likelihood of having severe anaphylaxis [44,45]. In fact, good asthma control may prevent life-threatening acute bronchospasm after ingestion of nuts, although there may be little effect on the severity of other symptoms of anaphylaxis such as pharyngeal edema [46].

This study has several potential limitations. First, we used administrative data, which are susceptible to coding errors and diagnostic misclassification. Therefore, we lack detailed information on the clinical criteria for diagnosing anaphylaxis. However, in Spain the medical societies recommend using the Guidelines of the European Academy of Allergy and Clinical Immunology [6]. Second, due to the nature of the national database, we did not have information that could be relevant such as complete information on etiology and risk factors/co-factors for anaphylaxis, detailed clinical presentation, serum (or plasma) tryptase, or pharmacologic treatments such as epinephrin injection. Third, our study did not include patients who were judged not to require hospitalization after an emergency department visit. Forth, in our study, we stratified by asthma and not by other atopic disease and clinical manifestation allergic rhinitis or/and eczema. We have chosen asthma because in Spain it is an important public health problem and has greater severity than other atopic diseases and clinical manifestations of allergic rhinitis and/or eczema. Furthermore, as commented before, there are few studies in our country and elsewhere that assess the association of anaphylaxis and asthma and the results have been contradictory. However, future investigations should focus on the relationship of anaphylaxis with other atopic disease and clinical manifestation allergic rhinitis or/and eczema. Fifth, in our investigation we have not included anaphylaxis triggered by a “toxic effect of contact with venomous animals and plants” (ICD10 codes T63.xxx). The reason for this is that from 2016 to 2021, only four children in the entire database (0.16%) had a T63.xxx code in their diagnosis fields. Sixth, we also could not identify those children that suffered anaphylaxis triggered by allergen immunotherapy or latex, as no ICD10 codes were available to identify a patient with anaphylaxis triggered by these allergens. Regarding allergen immunotherapy, the incidence rates of severe systemic reactions are estimated to be probably <1% [47].

The strengths of this study include the use of a large, nationally representative database, with a 6-year study period, which provides the ability to study trends of anaphylaxis in Spain over time. In addition, we used rigorous methods to select the study population and meticulous data analysis to measure outcomes.

## 5. Conclusions

In summary, using nationally representative data, we found that the number of hospitalizations for anaphylaxis in children remained stable from 2016 to 2019, with a drop in 2020 and recovery in 2021. Anaphylaxis hospitalization was more likely in male children, and food was the most common cause, increasing over time. IHM was low and remained stable during the study period. On the other hand, the incidence of hospitalizations for anaphylaxis was higher in asthmatic children than in non-asthmatics, but there were no differences in the occurrence of severe anaphylaxis among them. Increased knowledge regarding the epidemiology of pediatric anaphylaxis in children may contribute to improve its management.

## Figures and Tables

**Table 1:** Characteristics of hospital admissions with a diagnosis of anaphylaxis among children in Spain, 2016–2021.

	2016	2017	2018	2019	2020	2021	Total	*p* Trend
N	431	421	439	432	346	504	2573	NA
Age, mean (SD)	6.9 (4.82)	7 (4.86)	6.35 (4.77)	6.94 (4.95)	6.78 (5.15)	6.68 (4.99)	6.77 (4.92)	0.396
0–5 years old, n (%)	189 (43.85)	189 (44.89)	214 (48.75)	189 (43.75)	159 (45.95)	238 (47.22)	1178 (45.78)	0.558
6–11 years old, n (%)	153 (35.5)	130 (30.88)	145 (33.03)	149 (34.49)	107 (30.92)	165 (32.74)	849 (33)	
12–17 years old, n (%)	89 (20.65)	102 (24.23)	80 (18.22)	94 (21.76)	80 (23.12)	101 (20.04)	546 (21.22)	
Boys, n (%)	280 (64.97)	245 (58.19)	255 (58.09)	250 (57.87)	225 (65.03)	305 (60.52)	1560 (60.63)	0.081
Anaphylactic reaction due to food, n (%)	292 (67.75)	260 (61.76)	282 (64.24)	270 (62.5)	244 (70.52)	383 (75.99)	1731 (67.28)	<0.001
Anaphylactic reaction due to serum, n (%)	15 (3.48)	14 (3.33)	17 (3.87)	8 (1.85)	10 (2.89)	14 (2.78)	78 (3.03)	0.600
Anaphylactic reaction due to drugs, n (%)	35 (8.12)	43 (10.21)	67 (15.26)	83 (19.21)	51 (14.74)	55 (10.91)	334 (12.98)	<0.001
Anaphylactic shock, unspecified, n (%)	91 (21.11)	105 (24.94)	72 (16.4)	73 (16.9)	42 (12.14)	53 (10.52)	436 (16.95)	<0.001
Invasive mechanical ventilation, n (%)	11 (2.55)	8 (1.9)	12 (2.73)	17 (3.94)	9 (2.6)	12 (2.38)	69 (2.68)	0.578
Noninvasive mechanical ventilation, n (%)	2 (0.46)	4 (0.95)	1 (0.23)	8 (1.85)	8 (2.31)	11 (2.18)	34 (1.32)	0.021
Admission to ICU, n (%)	29 (6.73)	55 (13.06)	49 (11.16)	59 (13.66)	44 (12.72)	36 (7.14)	272 (10.57)	0.001
IHM, n (%)	4 (0.93)	2 (0.48)	4 (0.91)	3 (0.69)	2 (0.58)	2 (0.4)	17 (0.66)	0.890
Admission to ICU or IHM, n (%)	31 (7.19)	55 (13.06)	49 (11.16)	60 (13.89)	45 (13.01)	37 (7.34)	277 (10.77)	0.001

ICU: Intensive Care Unit; IHM: In-hospital mortality; NA Not applicable.

**Table 2:** Characteristics of hospital admissions with a diagnosis of anaphylaxis among children with and without asthma in Spain, 2016–2021.

	Asthma	2016	2017	2018	2019	2020	2021	Total	*p* Trend	*p* “with Asthma” vs. “without Asthma”
N (%)	Yes	60 (13.92)	51 (12.11)	50 (11.39)	54 (12.5)	38 (10.98)	71 (14.09)	324 (12.59)	0.670	NA
	No	371 (86.08)	370 (87.89)	389 (88.61)	378 (87.5)	308 (89.02)	433 (85.91)	2249 (87.41)		
Age, mean (SD)	Yes	9.97 (4.04)	9.63 (3.39)	9.08 (4.45)	9.63 (3.81)	10.5 (3.87)	10.25 (3.5)	9.85 (3.84)	0.520	<0.001
	No	6.41 (4.76)	6.64 (4.92)	5.99 (4.7)	6.55 (4.98)	6.32 (5.1)	6.1 (4.95)	6.33 (4.9)	0.399	
0–5 years old, n (%)	Yes	10 (16.67)	6 (11.76)	13 (26)	6 (11.11)	5 (13.16)	6 (8.45)	46 (14.2)	0.194	<0.001
	No	179 (48.25)	183 (49.46)	201 (51.67)	183 (48.41)	154 (50)	232 (53.58)	1132 (50.33)	0.496	
6–11 years old, n (%)	Yes	27 (45)	25 (49.02)	20 (40)	31 (57.41)	15 (39.47)	42 (59.15)	160 (49.38)	0.194	<0.001
	No	126 (33.96)	105 (28.38)	125 (32.13)	118 (31.22)	92 (29.87)	123 (28.41)	689 (30.64)	0.496	
12–17 years old, n (%)	Yes	23 (38.33)	20 (39.22)	17 (34)	17 (31.48)	18 (47.37)	23 (32.39)	118 (36.42)	0.194	<0.001
	No	66 (17.79)	82 (22.16)	63 (16.2)	77 (20.37)	62 (20.13)	78 (18.01)	428 (19.03)	0.496	
Boys, n (%)	Yes	40 (66.67)	26 (50.98)	24 (48)	35 (64.81)	25 (65.79)	38 (53.52)	188 (58.02)	0.186	0.305
	No	240 (64.69)	219 (59.19)	231 (59.38)	215 (56.88)	200 (64.94)	267 (61.66)	1372 (61)	0.165	
Anaphylactic reaction due to food, n (%)	Yes	42 (70)	35 (68.63)	34 (68)	35 (64.81)	30 (78.95)	62 (87.32)	238 (73.46)	0.044	0.011
	No	250 (67.39)	225 (60.81)	248 (63.75)	235 (62.17)	214 (69.48)	321 (74.13)	1493 (66.39)	<0.001	
Anaphylactic reaction due to serum, n (%)	Yes	0 (0)	1 (1.96)	1 (2)	0 (0)	0 (0)	2 (2.82)	4 (1.23)	0.576	0.053
	No	15 (4.04)	13 (3.51)	16 (4.11)	8 (2.12)	10 (3.25)	12 (2.77)	74 (3.29)	0.616	
Anaphylactic reaction due to drugs, n (%)	Yes	4 (6.67)	2 (3.92)	6 (12)	6 (11.11)	2 (5.26)	3 (4.23)	23 (7.1)	0.413	0.001
	No	31 (8.36)	41 (11.08)	61 (15.68)	77 (20.37)	49 (15.91)	52 (12.01)	311 (13.83)	<0.001	
Anaphylactic shock, unspecified, n (%)	Yes	14 (23.33)	13 (25.49)	9 (18)	13 (24.07)	6 (15.79)	4 (5.63)	59 (18.21)	0.037	0.516
	No	77 (20.75)	92 (24.86)	63 (16.2)	60 (15.87)	36 (11.69)	49 (11.32)	377 (16.76)	<0.001	
Invasive mechanical ventilation, n (%)	Yes	2 (3.33)	2 (3.92)	0 (0)	5 (9.26)	1 (2.63)	0 (0)	10 (3.09)	0.054	0.630
	No	9 (2.43)	6 (1.62)	12 (3.08)	12 (3.17)	8 (2.6)	12 (2.77)	59 (2.62)	0.803	
Noninvasive mechanical ventilation, n (%)	Yes	0 (0)	2 (3.92)	0 (0)	2 (3.7)	2 (5.26)	0 (0)	6 (1.85)	0.152	0.374
	No	2 (0.54)	2 (0.54)	1 (0.26)	6 (1.59)	6 (1.95)	11 (2.54)	28 (1.24)	0.018	
Admission to ICU, n (%)	Yes	4 (6.67)	8 (15.69)	8 (16)	10 (18.52)	5 (13.16)	6 (8.45)	41 (12.65)	0.336	0.193
	No	25 (6.74)	47 (12.7)	41 (10.54)	49 (12.96)	39 (12.66)	30 (6.93)	231 (10.27)	0.004	
IHM, n (%)	Yes	1 (1.67)	0 (0)	1 (2)	0 (0)	0 (0)	0 (0)	2 (0.62)	0.555	0.918
	No	3 (0.81)	2 (0.54)	3 (0.77)	3 (0.79)	2 (0.65)	2 (0.46)	15 (0.67)	0.986	
Admission to ICU or IHM, n (%)	Yes	4 (6.67)	8 (15.69)	8 (16)	10 (18.52)	5 (13.16)	6 (8.45)	41 (12.65)	0.336	0.241
	No	27 (7.28)	47 (12.7)	41 (10.54)	50 (13.23)	40 (12.99)	31 (7.16)	236 (10.49)	0.006	

ICU: Intensive Care Unit; IHM: In-hospital mortality; NA Not applicable.

**Table 3:** Characteristics of hospital admissions with a diagnosis of anaphylaxis among children with and without asthma according to age groups in Spain, 2016–2021.

	Asthma	0–5 Years Old	6–11 Years Old	12–17 Years Old	*p*
Rate per 100,000 children per year	Yes	49.72	82.56	46.10	<0.001
	No	45.48	24.56	15.60	<0.001
Age, mean (SD)	Yes	3.67 (1.25)	8.63 (1.65)	13.91 (1.63)	<0.001
	No	2.22 (1.79)	8.3 (1.76)	14.02 (1.58)	<0.001
Boys, n (%)	Yes	30 (65.22)	97 (60.63)	61 (51.69)	0.186
	No	709 (62.63)	437 (63.43)	226 (52.8)	0.001
Anaphylactic reaction due to food, n (%)	Yes	39 (84.78)	119 (74.38)	80 (67.8)	0.081
	No	852 (75.27)	459 (66.62)	182 (42.52)	<0.001
Anaphylactic reaction due to serum, n (%)	Yes	0 (0)	1 (0.63)	3 (2.54)	0.257
	No	38 (3.36)	19 (2.76)	17 (3.97)	0.534
Anaphylactic reaction due to drugs, n (%)	Yes	0 (0)	12 (7.5)	11 (9.32)	0.109
	No	104 (9.19)	97 (14.08)	110 (25.7)	<0.001
Anaphylactic shock, unspecified, n (%)	Yes	7 (15.22)	28 (17.5)	24 (20.34)	0.708
	No	142 (12.54)	115 (16.69)	120 (28.04)	<0.001
Invasive mechanical ventilation, n (%)	Yes	0 (0)	5 (3.13)	5 (4.24)	0.370
	No	34 (3)	13 (1.89)	12 (2.8)	0.340
Noninvasive mechanical ventilation, n (%)	Yes	1 (2.17)	3 (1.88)	2 (1.69)	0.979
	No	16 (1.41)	6 (0.87)	6 (1.4)	0.568
Admission to ICU, n (%)	Yes	4 (8.7)	21 (13.13)	16 (13.56)	0.680
	No	105 (9.28)	61 (8.85)	65 (15.19)	0.001
IHM, n (%)	Yes	0 (0)	2 (1.25)	0 (0)	0.357
	No	8 (0.71)	5 (0.73)	2 (0.47)	0.852
Admission to ICU or IHM, n (%)	Yes	4 (8.7)	21 (13.13)	16 (13.56)	0.680
	No	109 (9.63)	62 (9)	65 (15.19)	0.002

*p* values for comparison by age groups. ICU: Intensive Care Unit; IHM: In-hospital mortality.

**Table 4:** Characteristics of hospital admissions with a diagnosis of anaphylaxis among children with and without asthma according to sex in Spain, 2016–2021.

	Asthma	Boys	Girls	*p*
Rate per 100,000 subjects per year	Yes	60.19	59.14	NA
	No	33.37	22.33	NA
Age, mean (SD)	Yes	9.51 (3.84)	10.32 (3.8)	0.060
	No	6.09 (4.74)	6.7 (5.11)	0.004
0–5 years old, n (%)	Yes	30 (15.96)	16 (11.76)	0.186
	No	709 (51.68)	423 (48.23)	0.001
6–11 years old, n (%)	Yes	97 (51.6)	63 (46.32)	0.186
	No	437 (31.85)	252 (28.73)	0.001
12–17 years old, n (%)	Yes	61 (32.45)	57 (41.91)	0.186
	No	226 (16.47)	202 (23.03)	0.001
Anaphylactic reaction due to food, n (%)	Yes	134 (71.28)	104 (76.47)	0.296
	No	938 (68.37)	555 (63.28)	0.013
Anaphylactic reaction due to serum, n (%)	Yes	4 (2.13)	0 (0)	0.087
	No	38 (2.77)	36 (4.1)	0.083
Anaphylactic reaction due to drugs, n (%)	Yes	13 (6.91)	10 (7.35)	0.880
	No	181 (13.19)	130 (14.82)	0.274
Anaphylactic shock, unspecified, n (%)	Yes	37 (19.68)	22 (16.18)	0.420
	No	221 (16.11)	156 (17.79)	0.298
Invasive mechanical ventilation, n (%)	Yes	5 (2.66)	5 (3.68)	0.601
	No	35 (2.55)	24 (2.74)	0.788
Noninvasive mechanical ventilation, n (%)	Yes	6 (3.19)	0 (0)	0.035
	No	16 (1.17)	12 (1.37)	0.673
Admission to ICU, n (%)	Yes	20 (10.64)	21 (15.44)	0.199
	No	138 (10.06)	93 (10.6)	0.677
IHM, n (%)	Yes	0 (0)	2 (1.47)	0.095
	No	8 (0.58)	7 (0.8)	0.541
Admission to ICU or IHM, n (%)	Yes	20 (10.64)	21 (15.44)	0.199
	No	141 (10.28)	95 (10.83)	0.675

*p* values for comparison by sex. ICU: Intensive Care Unit; IHM: In-hospital mortality; NA Not applicable.

**Table 5:** Multivariable analysis of the factors associated with in hospital mortality or admission to intensive care unit during hospital admission among children with a diagnosis of anaphylaxis in Spain, 2016–2021 according to asthma status.

	No Asthma	Asthma	Both
	OR (95% CI)	OR (95% CI)	OR (95% CI)
0–5 years old	1	1	1
6–11 years old	0.97 (0.68–1.38)	1.48 (0.43–5.16)	1.03 (0.74–1.43)
12–17 years old	1.48 (1.02–2.15)	1.22 (0.34–4.45)	1.39 (0.98–1.99)
Girls	0.97 (0.72–1.32)	1.87 (0.87–4.03)	1.05 (0.8–1.39)
Anaphylactic reaction due to drugs	2.03 (1.33–3.11)	1.77 (069–3.99)	1.95 (1.3–2.91)
Invasive mechanical ventilation	17.63 (9.5–32.73)	27.59 (4.77–159.64)	17.54 (9.87–31.15)
Noninvasive mechanical ventilation	3.59 (1.34–9.64)	42.5 (3.76–481.1)	4.9 (2.07–11.6)
2016	1	1	1
2017	2.17 (1.26–3.74)	2.2 (0.5–9.56)	2.25 (1.36–3.74)
2018	1.58(0.9–2.77)	3.64 (0.88–15.06)	1.79 (1.06–3)
2019	1.88 (1.09–3.25)	2.41 (0.58–10.09)	1.96 (1.18–3.26)
2020	1.97 (1.11–3.48)	2.26 (0.44–11.63)	2.03 (1.19–3.47)
2021	0.91 (0.5–1.67)	1.97 (0.45–8.58)	1.02 (0.58–1.78)
Asthma	NA	NA	1.31 (0.88–1.96)

OR: Odds Ratio; CI: Confidence Interval; NA: Not applicable.

## Data Availability

According to the contract signed with the Spanish Ministry of Health and Social Services, which provided access to the databases from the Spanish National Hospital Database (*Registro de Actividad de Atención Especializada. Conjunto Mínimo Básico de Datos*, Registry of Specialized Health Care Activities. Minimum Basic Data Set), we cannot share the databases with any other investigator, and we have to destroy the databases once the investigation has concluded. Consequently, we cannot upload the databases to any public repository. However, any investigator can apply for access to the databases by filling out the questionnaire available at https://www.sanidad.gob.es/estadEstudios/estadisticas/estadisticas/estMinisterio/SolicitudCMBD.htm (accessed on 20 May 2023). All other relevant data are included in the paper.

## Supplementary Materials

The following supporting information can be downloaded at: https://www.mdpi.com/article/10.3390/jcm12196387/s1, Table S1: ICD-10 diagnosis and procedures codes used in this investigation.
