# Supplementary material for: Trends and Hospital Outcomes in HOSPITAL Admissions for Anaphylaxis in Children with and without Asthma in Spain (2016–2021)
Source: J Clin Med. 2023 Oct 6;12(19):6387. doi: 10.3390/jcm12196387 (PMC10574011; doi:10.3390/jcm12196387)
Supplement: Supplementary file 1 [file jcm-12-06387-s001.zip › jcm-2638465-supplementary.pdf]

Table S1. ICD-10 diagnosis and procedures codes used in this investigation.

Variable	ICD-10
Anaphylaxis	T78.0xx, T78.2xxx, T80.5xxx, T88.6xxx
Anaphylactic reaction due to food, n (%)	T78.0xx
Anaphylactic reaction due to serum, n (%)	T80.5xxx
Anaphylactic reaction due to drugs, n (%)	T88.6xxx
Anaphylactic shock, unspecified, n (%)	T78.2xxx
Asthma, n (%)	J45
Noninvasive mechanical ventilation, n (%)	5A09357, 5A09457, 5A09557
Invasive mechanical ventilation, n (%)	5A1945Z, 5A1955Z, 5A1935Z
